# Fibrocartilage Stem Cells in the Temporomandibular Joint: Insights From Animal and Human Studies

**DOI:** 10.3389/fcell.2021.665995

**Published:** 2021-04-27

**Authors:** Yi Fan, Chen Cui, Peiran Li, Ruiye Bi, Ping Lyu, Yanxi Li, Songsong Zhu

**Affiliations:** ^1^State Key Laboratory of Oral Diseases, National Clinical Research Center for Oral Diseases, Department of Cariology and Endodontics, West China Hospital of Stomatology, Sichuan University, Chengdu, China; ^2^Hospital of Stomatology, Guanghua School of Stomatology, Sun Yat-sen University, Guangdong Province Key Laboratory of Stomatology, Guangzhou, China; ^3^State Key Laboratory of Oral Diseases, National Clinical Research Center for Oral Diseases, Department of Orthognathic and TMJ Surgery, West China Hospital of Stomatology, Sichuan University, Chengdu, China; ^4^State Key Laboratory of Oral Diseases, National Clinical Research Center for Oral Diseases, Department of Oral Implantology, West China Hospital of Stomatology, Sichuan University, Chengdu, China

**Keywords:** temporomandibular disorders, osteoarthritis, regeneration, condylar cartilage, mesenchymal stem cells

## Abstract

Temporomandibular disorders (TMD) are diseases involving the temporomandibular joint (TMJ), masticatory muscles, and osseous components. TMD has a high prevalence, with an estimated 4.8% of the U.S. population experiencing signs and symptoms, and represents a financial burden to both individuals and society. During TMD progression, the most frequently affected site is the condylar cartilage. Comprising both fibrous and cartilaginous tissues, condylar cartilage has restricted cell numbers but lacks a vascular supply and has limited regenerative properties. In 2016, a novel stem cell niche containing a reservoir of fibrocartilage stem cells (FCSCs) was discovered in the condylar cartilage of rats. Subsequently, FCSCs were identified in mouse, rabbit, and human condylar cartilage. Unlike mesenchymal stem cells or other tissue-specific stem/progenitor cells, FCSCs play a unique role in the development and regeneration of fibrocartilage. More importantly, engraftment treatment of FCSCs has been successfully applied in animal models of TMD. In this context, FCSCs play a major role in the regeneration of newly formed cartilage. Furthermore, FCSCs participate in the regeneration of intramembranous bone by interacting with endothelial cells in bone defects. This evidence highlights the potential of FCSCs as an ideal stem cell source for the regeneration of oral maxillofacial tissue. This review is intended to detail the current knowledge of the characteristics and function of FCSCs in the TMJ, as well as the potential therapeutic applications of FCSCs. A deep understanding of the properties of FCSCs can thus inform the development of promising, biologically based strategies for TMD in the future.

## Introduction

The temporomandibular joint (TMJ) is a unique articulation between the mandible and the temporal bone that consists of the temporal bone fossa, mandibular condyle, and articular disc (Ottria et al., 2018). The articular disc lies bilaterally between the glenoid fossa and condyle, separating the TMJ into upper and lower joint cavities. Characterized as a distinct hinge structure, the TMJ exhibits a complex range of movements, including sliding and rotation (Singh and Detamore, 2009; Bordoni and Varacallo, 2020). Among them, the lower joint compartment plays an essential role in rotational movement. The lubrication of synovial fluid and the glazed surface of condylar cartilage ensure smooth rotation and minimal abrasion of the TMJ (Vazquez et al., 2019). However, this condition is disrupted in the development of temporomandibular disorders (TMD) (Gauer and Semidey, 2015). The etiology of TMD is complex and multifactorial, including biological, environmental, emotional, and social triggers (Gauer and Semidey, 2015). However, the etiology of TMD progression is not fully delineated, and the primary pathology involves degeneration of the TMJ, known as osteoarthritis (OA) (Scrivani et al., 2008). The degenerative condition in the lower compartment of the TMJ directly affects the biomechanical properties of the cartilage and bone (Scrivani et al., 2008). Therefore, mandibular condylar cartilage is one of the most frequently affected sites (Iwasaki et al., 2017; Nickel et al., 2018). The current treatment strategies include non-surgical and surgical methods, which mainly relieve pain and improve the range of motion (Dimitroulis, 2018). These traditional therapies fail to recover the integrated structure of the TMJ. More importantly, due to the deficiency in nerves, blood vessels, and lymphatic cycling and the effect of persistent weight-bearing, there is a paucity of options to restore impaired condylar cartilage (Gauer and Semidey, 2015; Stoustrup and Twilt, 2015). Furthermore, unlike the hyaline cartilage covering the joint head in other synovial articulations, mandibular condylar cartilage is composed of fibrocartilage containing both fibrous and cartilaginous tissues, making regeneration more challenging (Huey et al., 2012). With advances in regenerative medicine, stem cell-based therapies have attracted much attention as an alternative way to repair diseased tissue in TMD (Cui et al., 2017; Jiang et al., 2020). Considering immune rejection, pathogen transmission, potential tumorigenesis, and host tissue engraftment, resident stem cells have profound advantages compared to exogenic stem cells (Huey et al., 2012; Centeno, 2014; Waskow, 2015). In this context, scientists have recently discovered a novel stem cell niche in the superficial zone of condylar cartilage, termed fibrocartilage stem cells (FCSCs) (Embree et al., 2016; Bi et al., 2020). FCSCs conform to the criteria of mesenchymal stem cells (MSCs) and have potential in cartilage and bone regeneration. This review outlines recent discoveries related to FCSCs, with a particular focus on their distinct characteristics and regulatory networks among species. An in-depth and comprehensive understanding of the properties of FCSCs can thus inform the development of biologically based strategies for TMD and other maxillofacial defects.

## Isolation of FCSCs From Animals and Human

**FIGURE 1:**
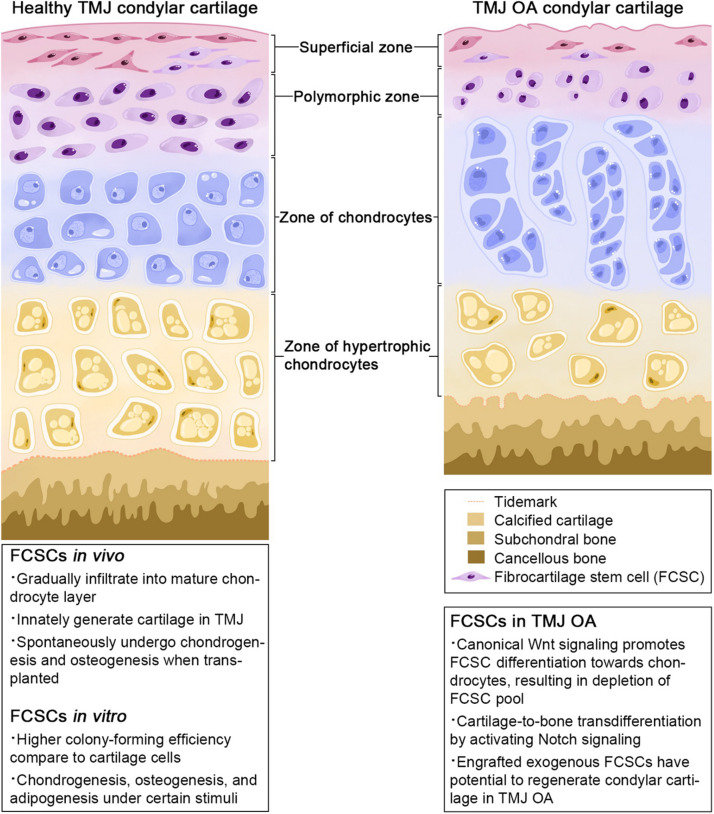
Zonal structure of TMJ condylar cartilage.

Of mesodermal origin, cartilage is a special connective tissue found in various sites throughout the body. Based on its composition and function, it comprises three types: hyaline cartilage, fibrocartilage, and elastic cartilage (Benjamin and Evans, 1990). Fibrocartilage contains a large number of collagen fibers and shows both the elasticity of cartilage tissue and the flexibility and toughness of fibrous tissue (Benjamin and Ralphs, 2004). Fibrocartilage has been discovered in the tendon, pubic symphysis, intervertebral discs, menisci, and TMJ (Benjamin and Ralphs, 2004). Histologically, TMJ fibrocartilage is divided into four layers: a fibrous superficial zone (SZ), a polymorphic zone, a zone of chondrocytes, and a zone of hypertrophic chondrocytes (Shibukawa et al., 2007) (Figure 1). A stem cell population, FCSCs, has been recently discovered in the SZ. FCSCs are mesenchymal-derived cells originating from condylar primordium blastema. The niche of FCSCs probably forms during the late period of the embryonic stage, participating in condyle development (Ruscitto et al., 2020). It is speculated that FCSCs exist over the span of a lifetime to maintain the homeostasis of mandibular condylar cartilage (Liang et al., 2016). To date, FCSCs in rats, mice, rabbits, and *Homo sapiens* have been identified by mesenchymal cell markers and location (Embree et al., 2016; Nathan et al., 2018; Bi et al., 2020; Ruscitto et al., 2020; Table 1).

**TABLE 1:** Characteristics and regulatory networks of FCSCs in animals and humans.

Species	Markers	Characteristics	Regulatory networks	Author and year
Rat	Positive: CD90, CD44, CD29, CD105, CD146 Negative: CD45, CD79a, CD11b	Reside in the SZ; Chondrogenic, osteogenic, and adipogenic differentiation capacity *in vitro*; High colony formation ability compared to that of cartilage cells; Spontaneous osteogenesis and chondrogenesis when transplanted *in vivo*	Wnt: deplete FCSCs pool and enhance chondrogenesis Notch: promote differentiation of FCSCs into chondrocytes and osteoblasts SOX9: induce chondrogenesis of FCSCs	[Bibr B20][Bibr B42][Bibr B50] Bi et al., 2020
Mouse	α-SMA+ cells in the SZ Notch+ cells during embryonic development	Reside in the SZ and gradually infiltrate into the mature chondrocyte layer	Notch: participate in TMJ morphogenesis and cartilage-to-bone transdifferentiation in TMJ OA	[Bibr B20][Bibr B50]
Human	Positive: CD44, CD73, CD90, CD105 Negative: CD29, CD34, CD45	Spindle-shaped cells; Comparable propagation ability, apoptosis rate, and colony forming efficiency to hOMSCs; Weak migration capability; Chondrogenesis under chondrogenic induction *in vivo*	SOX9: induce chondrogenesis of FCSCs	Bi et al., 2020
Rabbit	N/A	N/A	SOST: maintain the FCSC pool	Embree et al., 2016

*FCSCs, fibrocartilage stem cells; SZ, superficial zone; hOMSCs, human orofacial bone marrow-derived mesenchymal stem cells; SOX9, Sry-related HMG box-9; TMJ, temporomandibular joint; OA, osteoarthritis; SOST, sclerostin.*

Fibrocartilage stem cells were first isolated by Embree et al. (2016) from the rat TMJ. In this study, condyles were dissected from Sprague–Dawley rats at 6–8 weeks of age, followed by a 15-min digestion process containing 4 mg/ml dispase II at 37°C to separate the SZ. Then, the SZ was continuously digested in 4 mg/ml dispase II and 3 mg/ml collagenase I to harvest all nucleated cells. Cellular suspensions were cultured in basal medium containing 55 mM 2-mercaptoethanol. Rat FCSCs were confirmed by surface markers of MSCs. They were positive for CD90, CD44, CD29, CD105, and CD146 but negative for CD45, CD79a, and CD11b (Embree et al., 2016).

Subsequently, mouse FCSCs in the TMJ condyle were identified by using the stem cell label α-SMA (Embree et al., 2016). α-SMA is commonly known as a myofibroblast marker and has been implicated in organ fibrosis (Bhowmick et al., 2004; Darby and Hewitson, 2007). Lee et al. proved that human MSCs have a stepwise process of fibroblast differentiation *in vitro*. By analyzing α-SMA expression, they found that human MSCs express α-SMA under connective tissue growth factor (CTGF) and transforming growth factor (TGF)-β1 stimulation. In this study, cells expressing α-SMA were deemed to present a myofibroblast phenotype (Lee et al., 2010). In addition, α-SMA expressed in hypertrophic chondrocytes was one of the indicators of fibrosis of condylar cartilage in TMJOA progression (Wang et al., 2012). Furthermore, Zhang et al. (2019) found that the percentage of α-SMA^+^ cells was positively correlated with Mankin scores of OA. These studies focused on the expression of α-SMA in the layer of chondrocytes and hypertrophic chondrocytes. The role of α-SMA as a typical skeletal stem/progenitor cell marker has been recently discovered. α-SMA was originally found in smooth muscle cells and vascular pericytes as a cell marker. As the osteogenic potential of pericytes was proven (Doherty et al., 1998), scientists assumed that pericytes have similar characteristics to MSCs. After that, Shi and Gronthos (2003) labeled α-SMA in MSCs derived from bone marrow (BMMSCs) and dental pulp stem cells (DPSCs) and found that α-SMA^+^ cells have characteristics similar to those of smooth muscle cells and pericytes. Furthermore, Grcevic et al. (2012) identified α-SMA^+^ cells as typical skeletal progenitor cells responsible for new bone formation and fracture healing. Hence, Embree et al. performed a lineage-tracing experiment using α-SMACreERT2/Ai9 transgenic mice as an indicator of progenitor cells in condylar cartilage. α-SMACreERT2/Ai9 mice were injected with tamoxifen at postnatal day 16 (P16) and sacrificed after 2 and 15 days. The results suggested that α-SMA^+^ cells were concentrated in the SZ 2 days after tamoxifen administration and increased in condylar cartilage after 15 days. Moreover, it has been verified that the extracellular matrix of FCSCs in the SZ is different from that of mature chondrocytes in condylar cartilage. FCSCs do not express aggrecan and collagen II (Col II), proteins secreted from mature chondrocytes, but are surrounded by lubricin and collagen I (Col I) (Embree et al., 2016). Therefore, the SZ may provide a niche for undifferentiated cells, and these α-SMA^+^ cells in the SZ are able to differentiate into chondrocytes. In addition, Ruscitto et al. found that Notch signaling in FCSCs participated in TMJ morphogenesis, indicating that Notch-Venus reporter mice are an alternative choice to label FCSCs in mouse condylar cartilage during embryonic development (Nathan et al., 2018). However, mouse FCSCs have not been successfully isolated and cultured *in vitro* due to their small number in the mouse TMJ. Therefore, further research is required to optimize the isolation method to harvest mouse FCSCs.

More recently, we cultivated human FCSCs from condylar pieces harvested from patients with condylar comminuted fractures (Bi et al., 2020). Owing to the large volume of human tissue, the superficial zone of the condylar cartilage was cut into 1 mm × 1 mm pieces before digestion. The following enzymatic digestion method was similar to that used for rat FCSC isolation. Then, the surface markers of the cultured cells were identified by flow cytometry. The results showed that human FCSCs were positive for CD44, CD73, CD90, and CD105 but negative for CD29, CD34, and CD45, consistent with the cellular surface markers of rodent FCSCs (Embree et al., 2016; Bi et al., 2020). According to the report from Dominici et al. (2006), MCSs express CD73, CD90, and CD105 but are negative for CD34, CD45, CD14, CD11b, CD19, and CD79α. Both human FCSCs and rat FCSCs expressed classical Dominici MSC markers, such as CD44, CD90, and CD105. Comparatively, human FCSCs were negative for CD34 and CD45 (Bi et al., 2020), while rat FCSCs lacked expression of CD45, CD11b, and CD79α (Embree et al., 2016). Notably, CD29 was found to be positive in rat FCSCs but negative in humans. Moreover, human FCSCs are positive for CD73, but this was not verified in rat FCSCs. Whether there are other diverse surface markers among species remains to be determined. Further analysis, such as using single-cell technology, may unravel the specific markers of FCSCs and help to dissect the desired cell population and generate novel mouse models for directly targeting FCSCs.

## Characteristics of FCSCs Under Physiological Conditions

### Proliferation

A colony-forming assay was performed to evaluate the proliferation rates of rat FCSCs *in vitro*, showing that rat FCSCs formed sixfold more colonies than donor-matched cartilage cells (Embree et al., 2010, 2016). Canonical Wnt signaling was closely tied to the proliferative activity of rat FCSCs. By adding sclerostin (SOST), an inhibitor of Wnt signaling, the proliferation rates of FCSCs were significantly reduced (Embree et al., 2016). In *H. sapiens*, FCSCs show self-renewal ability and are able to maintain their spindle-shaped morphometry, proliferation, apoptosis, and senescence abilities after expansion. They have comparable propagation ability, apoptosis rates, and colony-forming efficiency to orofacial bone marrow-derived mesenchymal stem cells (OMSCs) (Bi et al., 2020).

### Differentiation

Fibrocartilage stem cells possess many *in vitro* features of MSCs, including clonogenicity and multipotential differentiation capacity. Under stimulation, FCSCs can differentiate into osteogenic, chondrogenic, and adipogenic cells. When rat FCSCs were cultured in chemically defined media, over 87% of individual colonies exhibited heterogeneous differentiation potential (22.5% trilineage, 64.5% bilineage) (Embree et al., 2016). Human FCSCs, comparing to human OMSCs, had a comparable adipogenic potential but a reduced osteogenesis potential during multi-lineage differentiation *in vitro*. Increasing evidence has revealed that multiple signaling pathways control FCSC differentiation *in vitro*, such as the canonical Wnt and Notch signaling pathways. After transfection of β-catenin, FCSCs exhibited decreased expression of cartilage-related transcription factors, such as *sox5, sox6, and sox9* (Embree et al., 2016). Another study found that SOX9 was more highly expressed in human FCSCs than in cartilage cells (Embree et al., 2010, 2016) and other mesenchymal stem cells (Bi et al., 2020; Jiang et al., 2020). When SOX9 expression was interfered with, FCSCs were unable to form well-organized cartilaginous tissue under chondrogenic induction (Bi et al., 2020). Furthermore, Ruscitto et al. (2020) revealed that Notch signaling plays a key role in promoting FCSC differentiation into chondrocytes and osteoblasts but not adipogenic cells *in vitro*.

Fibrocartilage stem cells also present multipotential capacity *in vivo*. FCSCs traced by the skeletal stem/progenitor cell marker α-SMA in adult mice showed that the α-SMA^+^ cells in the SZ gradually infiltrated into the mature chondrocyte layer and expressed the chondrocyte marker Col II. This result indicated that FCSCs have the potential to differentiate toward chondrocytes to maintain the homeostasis of condylar cartilage under physiological conditions (Embree et al., 2016). Wnt signaling is also involved in the chondrogenesis of FCSCs. The downstream Wnt mediator β-catenin is expressed in mature chondrocytes but not in the SZ, implicating that Wnt activity is restrained in the SZ. Notably, SOST suppressed FCSC proliferation as previously noted, yet SOST knockout mice showed depletion of the FCSC pool (Embree et al., 2016). It has been speculated that in SOST knockout mice, Wnt signaling was enhanced significantly in the SZ and thus induced the differentiation of FCSCs toward chondrocytes, emphasizing the function of Wnt signaling in directing FCSC fate.

Moreover, recent research has confirmed the strong osteogenic and chondrogenic capability of exogenic FCSCs. Subcutaneously transplanted rat FCSCs with collagen sponges could form cartilaginous-like tissue, which then gradually transformed into transitional tissue (bone, cartilage, and osteoclast-mediated tissue) and resulted in well-organized trabecular bone-like tissue (Embree et al., 2016). Collectively, these results indicate that FCSCs are able to differentiate into multiple cell lineages and spontaneously recapitulate endochondral ossification when transplanted *in vivo* (Yang et al., 2014). Human FCSCs have different fates than rat FCSCs in xenograft models. Human FCSCs need chondrogenic induction before transplantation; otherwise, they are not able to undergo a similar process to rat FCSCs (Bi et al., 2020).

The regulation of stem cell populations is tightly controlled by the local microenvironment according to the requirements of the host tissue (Fuchs and Segre, 2000; Bianco and Robey, 2001). When injected into mandibular condylar cartilage defects, FCSCs spontaneously formed cartilage, and no bone-forming process was observed during follow-up (Bi et al., 2020). FCSCs undergo a chondrogenic differentiation fate in the microenvironment of the lower joint compartment. However, ectopic xenografts of rat FCSCs begin to form bone-like tissue after 4 weeks of observation, indicating that FCSCs have the tendency to undergo hypertrophy to form bone in a subcutaneous environment (Embree et al., 2016). These distinct differentiation patterns highlight the importance of the microenvironment in FCSC fate decisions. To date, the detailed modulatory mechanisms by which the microenvironment affects FCSC fate have not been fully characterized, but this could be a target for researchers in future studies of FCSCs.

### Migration

The analysis of FSCS migration is somewhat limited. By performing the scratch wound healing assay, we found that human FCSCs presented a weaker migration capability than OMSCs (Bi et al., 2020). The migratory ability of FCSCs *in vivo* and whether these endogenous stem cells can be recruited to defect sites remain to be determined.

### Trophic and Immunomodulatory Functions

Increasingly, the mechanisms underlying the therapeutic effects of MSCs are attributed to the secretion of trophic factors, particularly extracellular vesicles (EVs) (Meirelles Lda et al., 2009). EVs are cell-derived membrane-bound nanoparticles that play an important role in the maintenance of biophysiological homeostasis as well as cellular, physiological, and pathological processes (Yáñez-Mó et al., 2015). EVs have significant diagnostic and therapeutic potential. MSCs, as prolific producers of EVs, have recently attracted much attention (Baglio et al., 2012; Liang et al., 2014). Of note, exosomes, one type of EV with a 40–100 nm diameter (Raposo and Stoorvogel, 2013), secreted by MSCs are found to have a great effect on the treatment of OA (Zhu et al., 2017). In TMJOA treatment, Zhang et al. (2016, 2019) administered exosomes isolated from human embryonic stem cell-derived MSCs to treat TMJOA and found that they could promote TMJ repair. However, the trophic function of FCSCs is still not well characterized. FCSCs have the potential to secrete trophic factors, particularly exosomes, which are crucial for therapeutic function. Moreover, a previous report compared exosomes secreted by synovial membrane MSCs and induced pluripotent stem cell-derived MSCs in the treatment of OA in the knee joint. Both exosomes could attenuate OA, but the latter had a better therapeutic effect (Zhu et al., 2017). Because exosomes secreted by different types of MSCs show distinct regenerative capacities, it is crucial to investigate the trophic function of FCSCs as well as their cell-specific properties of trophic factors in TMJOA treatment.

Moreover, increasing evidence indicates that MSCs play an immunomodulatory role primarily through the release of EVs and paracrine factors (Spees et al., 2016; Li and Hua, 2017). Previous reports have found that MSC-derived exosomes and microparticles play an anti-inflammatory role independently to modulate T and B lymphocytes in inflammatory arthritis (Cosenza et al., 2018). Whether FCSCs have immunomodulatory capacity during condylar cartilage regeneration under pathological conditions remains to be determined. Scholars have found that FCSCs can secrete VEGF-A in a paracrine manner *in vitro* (Nathan et al., 2018). This may help to explain how FCSCs organize the hematopoietic microenvironment *in vivo* (Embree et al., 2016), highlighting the possibility of interactions of FCSCs and surrounding cells in a paracrine manner. Further study of the trophic and immunomodulatory functions of FCSCs is needed.

### Differences Between FCSCs and BMMSCs

Compared with BMMSCs, FCSCs express similar cell surface markers, including CD90, CD44, CD29, CD105, and CD146, but lack leukocyte markers, such as CD45, CD79a, and CD11b (Soleimani and Nadri, 2009; Robey et al., 2021). As noted above, they show heterogeneous differentiation potential similar to that of BMMSCs *in vitro* (Embree et al., 2016). It is important to note that FCSCs show distinct progress of osteogenesis when transplanted onto the dorsum of athymic nude mice. FCSCs formed cartilaginous-like tissue first and then transformed into bone-like tissue, while BMMSCs directly formed bony tissue without cartilaginous tissue transition (Embree et al., 2016). Notably, chondrogenically precultured BMMSCs could form unstable cartilage with hypertrophy, vascular invasion, and terminal matrix calcification (Pelttari et al., 2006). In general, compared to BMMSCs, FCSCs have innate chondrogenic capacity in the context of transplantation.

### Comparison Among FCSCs and Other Fibrocartilage Tissue-Derived Stem Cells

While FCSCs are stem cells in the fibrocartilage of the TMJ, there are various stem cells that can be isolated from fibrocartilage in other organs, including meniscus-derived mesenchymal stem cells (MMSCs), annulus fibrosus-derived stem cells (AFSCs), and tendon-derived stem cells (TDSCs). MMSCs are isolated from avascular zone of meniscus, which express MSCs surface markers, such as CD44 and CD90 (Gui et al., 2015; Huang et al., 2016). When compared to BMMSCs, MMSCs showed a stronger chondrogenesis *in vitro* and a better repair of damaged meniscus *in vivo* (Ding and Huang, 2015). Similar to FCSCs, MMSCs preferentially differentiate into chondrocytes (Huang et al., 2016). Annulus fibrosus is a fibrocartilaginous tissue in intervertebral disc (Liu et al., 2014). AFSCs express common MSCs surface markers, including CD29, CD44, and CD166 (Liu et al., 2014; Guo et al., 2018). They could form a hierarchical structure approximating native AF tissue (Chu et al., 2018; Zhou et al., 2021). TDSCs express a similar surface marker with MSCs, including CD44 and CD90 (Bi et al., 2007; Liu et al., 2018). Unlike FCSCs and MMSCs, TDSCs preferentially differentiated into tenocyte-like cells but not chondrocytes (Guo et al., 2016), emphasizing the potential of TDSCs in repairing bone-tendon junction, a fibrocartilaginous structure in tendon (Benjamin and Ralphs, 1998; Qin et al., 2020). These data suggest that stem cells originated from certain fibrocartilage tissue may have their unique differentiation signature, possibly reflecting their site of origin.

### Interactions Between FCSCs and Human Umbilical Vein Endothelial Cells

Angiogenesis is a tightly regulated process involved in the growth and repair of bone tissue. Several studies have verified that human umbilical vein endothelial cells (HUVECs) can indirectly regulate BMMSCs *via* angiocrine factors (Villars et al., 2000; Zhu et al., 2020). However, Nathan et al. proved that the secreted factors of HUVECs were not sufficient to stimulate FCSCs *in vitro*. Only when in direct contact with HUVECs were the osteogenic transcription factors of FCSCs markedly upregulated (Nathan et al., 2018). Furthermore, FCSCs in turn support angiogenesis. Vascular endothelial growth factor A (VEGF-A), a cytokine promoting HUVEC proliferation, was highly expressed in FCSCs when cultured *in vitro*. The number of HUVECs significantly increased when cultured in FCSC-conditioned medium (Nathan et al., 2018). However, some studies reported different results. The fibrinogen gel bead angiogenesis assay (FIBA) suggested that direct interactions between FCSCs and HUVECs impeded angiogenesis (Nathan et al., 2018). Therefore, more research is needed to explore whether other FCSC-derived factors affect HUVECs in addition to their paracrine function through VEGF-A.

## Therapeutic Application

### Treatment of Temporomandibular Joint Osteoarthritis

Temporomandibular joint OA is one of the most severe subtypes of TMD due to degeneration of various hard and soft tissues, including cartilage degeneration, viscous synovial fluid accumulation, and osteophyte formation (Rando and Waldron, 2012; Bechtold et al., 2016; Ibi, 2019). Existing treatments for TMJ OA mainly focus on pain relief and functional rehabilitation. There is difficulty in recovering the physiological morphology and function of condylar cartilage. Therefore, clinical therapy is urgently needed to restore the TMJ structure and regenerate defects. Residing in the SZ of cartilage, FCSCs harbor multilineage differentiation potential and participate in cartilage formation, implying their potential in repairing defects in TMJ OA. It was discovered that the application of an exogenous Wnt inhibitor could repair and regenerate injured fibrocartilage by maintaining the FCSC pool and regulating FCSC differentiation. Embree et al. arranged SOST injection into a rabbit TMJ OA model and found that the condyles had mild surface irregularities after SOST administration. The contralateral PBS-treated condyles displayed severe surface irregularities and had significantly higher Osteoarthritis Research Society International (OARSI) recommended macroscopic scores. Moreover, SOST treatment led to a significantly greater number of cells surviving in the SZ, indicating that Wnt inhibitors could protect FCSCs from depletion and improve the morphology of condylar cartilage in the progression of TMJ OA (Embree et al., 2016).

Accelerated cartilage-to-bone transformation is one of the main causes of condylar bone reconstruction in TMJ OA (Liu et al., 2015). Ruscitto et al. discovered that Col II/Runx2 double-positive cells located at the cartilage/bone interphase did not express Notch1 in the normal mandibular condyle. However, after local delivery of TNF-α to induce TMJ OA, Col II/Runx2^+^ cells appeared in the SZ and were positive for Notch1, implying that Notch1 mediated FCSCs cartilage-to-bone transformation in the setting of TMJ OA (Ruscitto et al., 2020). Therefore, Notch inhibitors offer promising therapeutic potential in the treatment of TMJ OA by maintaining the morphology of the condyle. The Notch inhibitors γ-secretase inhibitor IX and N-[N-(3,5-difluorophenacetyl-L-alanyl)]-(S)-phenylglycine t-butyl ester (DAPT) significantly reduced the expression of *Notch 1, Runx2*, and *Ocn* in FCSCs and suppressed osteogenesis of FCSCs *in vitro* (Ruscitto et al., 2020). Although the effect of Notch inhibitors on FCSCs has not been evaluated *in vivo*, increasing evidence suggests the potential role of Notch inhibitors in the treatment of TMJ OA by targeting FCSCs (Luo et al., 2018).

More recently, we transplanted exogenous FCSCs into a TMJ defect rat model to assess their function in cartilage repair (Bi et al., 2020). After 4 weeks, engrafted FCSC lineages could be observed in the SZ, polymorphic zone, and zone of chondrocytes. Under gross observation, the condylar surface of the defect sites was smoother in the FCSC-treated group than in the vehicle-treated group. The International Cartilage Regeneration and Joint Preservation Society (ICRS) score and modified Mankin score were utilized to evaluate the effectiveness of FCSC treatment, showing that FCSC treatment improved the arrangement of cartilage structures. These results indicate that FCSCs are an optimal stem cell source facilitating TMJ cartilage repair *in vivo.*

### Regeneration of Maxillofacial Bone

Previous studies have demonstrated that the interactions of FCSCs and HUVECs could promote osteogenic differentiation of FCSCs *in vitro*. Researchers further generated a mouse model with critical-size defects in the calvaria to mimic the vascularized bone niche and found that FCSC transplantation directly formed bone-like tissue in the defect region (Nathan et al., 2018). FCSCs were able to differentiate and form *de novo* bony tissue that expressed OCN. In addition, the neovasculature localized at the periphery of the FCSC engraftment area was CD31^+^, suggesting that FCSC integration was coupled with endothelial cell recruitment (Embree et al., 2016; Nathan et al., 2018). Notably, dorsum-transplanted FCSCs regenerated cartilage before calcification, which differed from the direct formation of bone-like tissue in calvarial defects. Scholars speculated that the microenvironment may contribute to FCSC fate decisions. In the ectopic xenograft model, the innate chondrogenic capacity of FCSCs dominated the regeneration process, while the osteogenesis of FCSCs observed in the vascularized bone defect may rely on FCSC–HUVEC interactions. At present, the mechanisms modulating FCSC differentiation toward chondrogenesis and osteogenesis *in vivo* remain uncertain, and further investigation is warranted.

## Conclusion

Fibrocartilage stem cells, a novel stem cell population, have been recently identified in the condylar cartilage of animals and humans. Under physiological conditions, FCSCs play an indispensable role in the development and homeostasis of condylar cartilage. They present clonogenicity and multipotency, sharing similar *in vitro* properties with MSCs. Recent attention has been focused on the regulatory mechanisms of FCSCs, implying their distinct characteristics during development. However, whether FCSCs have a unique signature compared to other resident dental MSC populations remains to be determined. More importantly, endogenous and exogenous FCSCs hold enormous promise in cartilage and bone repair and regeneration in pathologic states. The mechanism may involve the Wnt and Notch signaling pathways, but the precise regulatory networks have not been fully clarified. There is still controversy regarding the differentiation process of FCSCs when transplanted in different sites; thus, it is of crucial importance to perform a more comprehensive analysis of *in vivo* changes as well as the interaction between FCSCs and their microenvironment. In summary, understanding the functions and regulatory mechanisms of FCSCs will aid the establishment of FCSC-based strategies for cartilage and bone regeneration.

## Author Contributions

YF, CC, PLi, PLy, YL, and RB collected the literature and drafted the manuscript. YF, RB, and SZ supervised the procedures and approved the manuscript. All authors gave their final approval and agreed to be accountable for all aspects of the work.

## Conflict of Interest

The authors declare that the research was conducted in the absence of any commercial or financial relationships that could be construed as a potential conflict of interest.
